# Social and emotional skills of students at Child Protection and Welfare Bureau Gujranwala, Pakistan

**DOI:** 10.1186/s41155-026-00401-5

**Published:** 2026-06-17

**Authors:** Sahrish Nayab, Muhammad Sarwar, Khawaja Hisham-Ul-Hassan, Mehmood-Ul- Hassan, Asma Abdul Aziz

**Affiliations:** 1 Faculty of Arts and Humanities, Superior University, Lahore, Pakistan; 2 Department of Economics and Commerce, Superior University, Lahore, Pakistan; 3 Department of English Language and Literature, The Shaikh Ayaz University, Shikarpur, Sindh, Pakistan; 4 School of Languages Civilization & Philosophy, Universiti Utara, Malaysia, Malaysia

**Keywords:** Social skills, Emotional skills, Child, Child protection

## Abstract

**Background:**

This study analyzes the social and emotional skills of students at the *Child Protection and Welfare Bureau*,* Gujranwala (CP&WB*). The CP&WB in Gujranwala, Pakistan, is an important organization that safeguards children’s rights and well-being in precarious circumstances. The development of children’s social and emotional skills in protective institutions such as CP&WB Gujranwala is crucial, as these have a direct impact on their overall growth, well-being, and learning outcomes.

**Objectives:**

This study aimed to (a) analyze the levels of social and emotional skills among the students enrolled at *CP&WB Gujranwala*, (b) examine the difference in social and emotional skills levels between students with shorter institutional stay (less than six months) and those with longer institutional stay (six months and more) at *CP&WB Gujranwala*, and (c) investigate the relationship between institutional stay duration and students’ social and emotional skills at *CP&WB Gujranwala.*

**Methodology:**

The research adopts a sequential exploratory mixed-method design, combining in-depth interviews with teachers using a semi-structured interview guide and a structured quantitative survey of students with an adapted social-emotional tool. This study encompasses all (7) teachers and (105) students as a universal sample. Validity and reliability were assessed. Thematic analysis for the qualitative part and descriptive (mean, SD, frequency) and inferential statistical (t-test, Pearson correlation) analyses were conducted for the quantitative part of the study.

**Results:**

Findings indicate that an extended institutional stay is associated with higher levels of students’ social and emotional competencies, with significant correlations linking longer durations to improved self-regulation, empathy, and positive social interactions. Quantitative analysis highlighted notable progress in skills such as sharing, self-expression, and relationship-building, while thematic analysis of teacher interviews revealed variations in students’ adaptation, highlighting personal backgrounds as influencing factors.

**Conclusion:**

The study’s results underscore the *CP&WB*’s role in fostering social and emotional growth through structured support. Statistical analyses revealed highly significant associations between longer institutional stay duration and social and emotional skills. This research contributes original insights into the effectiveness of institutional interventions for marginalized youth, suggesting that prolonged, supportive stays enhance students’ social and emotional well-being.

## Introduction

Pakistan is a country with a majority of Muslim residents and a lively mix of ethnicities and cultures. English is the official language, with Urdu also being recognized as the national language and widely spoken and understood throughout the country. In cities, nuclear families are becoming more common as traditional joint family systems are being replaced, especially with the rise of modernity and urbanization. Around 56.5 million people in Pakistan are currently living in poverty, which accounts for about 25% of the total population of 226 million. Less than 25% of the 80 million children in the country have access to basic necessities, especially in urban areas. Even though Pakistan offers free and compulsory education for children between the ages of five and sixteen, the country has the second-highest number of out-of-school children in the world, with a concerning 44% of school-aged children not enrolled in school. The rate of students dropping out is notably high, as 44% of boys and 56% of girls quit school before finishing fifth grade (Hébert, 2022; Hoemann et al., 2021; Lodhi et al., 2021; UNICEF, 2024). In Pakistan, the education system consists of public and private schools, along with madrasas, but struggles with problems such as outdated curricula and a lack of focus on overall development. Despite promises of government reform, challenges like overcrowded classrooms and outdated teaching methods, as seen in public schools, continue to exist (Khan, 2018; Wilgus, 2019).

The *CP&WB* in *Gujranwala*, Pakistan, is an important organization that safeguards children’s rights and well-being in precarious circumstances. Around 200 children are accommodated at *CP&WB Gujranwala*, where the organization collaborates with different agencies and stakeholders to enhance child welfare programs and services (Apeli & Kisimbii, 2020). As per the United Nations Convention on the Rights of the Child, a child is described as an individual who is below the age of eighteen (Lansdown & Vaghri, 2022). The concept of “child protection” includes all efforts to prevent, address, and handle instances of abuse, exploitation, and violence towards children (Garcia et al., 2024; Lansdown & Vaghri, 2022). *CP&WB* has a specific role in caring for and rehabilitating abandoned children, following government policies to guarantee child safety and welfare (Nayab et al., 2023).

In South Asian cultures, like in Pakistan, children are frequently taught to display deference towards their elders and uphold a compulsory level of respect. Yet, this societal expectation can also lead to instances of child maltreatment that are tolerated in certain environments, resulting in decreased chances of reporting because of feelings of disgrace, embarrassment, and concerns about legal consequences (Chaudhuri, 2017). Findings from Shekhar and Rai (2025) and UNICEF (2020, 2025) show that there is a high prevalence of physical and psychological abuse in South Asia, resulting in serious outcomes like being kicked out of school, being without a home, being put in jail, and even turning to prostitution. South Asian countries are now focusing more on child protection measures due to growing awareness of child safety concerns, aiming to address the various dangers children are exposed to (Hyder & Mac Veigh, 2007).

Neglected and destitute children living under diverse circumstances, such as poverty, neglect, abuse, or institutional care, face many difficulties in their social and emotional development. These circumstances affect their behavior, emotional control, and relationships with others. Research shows that social and emotional skills are learned behaviors that are acquired through interpersonal experiences with peers, caregivers, and parents (Greenberg, 2023; Payton et al., 2000). Institutionalized children face many difficulties, such as emotional regulation problems, trust issues, limited social interaction, and reduced empathy, due to the absence of these experiences (Kaur et al., 2018; Lamm et al., 2018; Li et al., 2019). In the South Asian and Pakistani contexts, children living in vulnerable and institutional environments face difficulties with emotional regulation, social relations, and psychological well-being (Najmussaqib & Mushtaq, 2023; Sharma et al., 2025; Zaman et al., 2024). Similarly, intervention research conducted in Pakistani schools has demonstrated that social and emotional functioning can be enhanced through a structured support system (Najmussaqib et al., 2024). These studies further emphasize the importance of context-specific research.

The current study used CASEL’s social and emotional competency domains as an organizing framework for categorizing and interpreting social and emotional skills. The CASEL framework defines SEL as the process through which individuals acquire the attitudes, information, and abilities needed to handle emotions, establish and accomplish goals, show empathy, create healthy relationships, and make appropriate choices (Collaborative for Academic and Emotional Learning, 2021, 2023). The five main skills in the CASEL framework: self-awareness, self-management, social awareness, relationship skills, and responsible decision-making, are essential for evaluating the development of social and emotional skills. The CASEL framework is especially useful for children living in institutional care because it views social and emotional skills as not innate but as can be cultivated through targeted interventions and practice, such as social and emotional skills training programs, which have proven beneficial, particularly for individuals facing social and emotional challenges (Collaborative for Academic and Emotional Learning, 2023; Domitrovich et al., 2025; Payton et al., 2000). Additionally, the interplay between social skills and emotional intelligence plays a significant role in navigating complex social environments (Wang et al., 2024). As outlined by research, emotional development pertains to the progression of equipping students with the capacity to sensibly comprehend themselves, their emotions, their sentiments, and their environment, while also cultivating the ability to interact with others in a considerate manner (Greenberg, 2023; Jones & Doolittle, 2017). In difficult settings like the Child Protection and Welfare Bureau in Punjab, developing these skills can greatly influence students’ well-being. The researcher used CASEL’s four competencies (self-awareness, social awareness, self-management, and relationship skills) to organize the social and emotional construct.

The development of children’s social and emotional skills in protective institutions such as *CP&WB Gujranwala* is crucial, particularly because children in such settings often experience social vulnerability, including neglect, abuse, and disrupted caregiving, which can adversely affect their emotional regulation and interpersonal functioning. These skills have a direct impact on their overall growth, well-being, and learning results, according to Bjugstad et al.(2025); Chaikovska et al. (2023), and Ross and Heath (2002). Social skills encompass various behaviors that aid in achieving social goals, including communication (verbal, nonverbal, written, and visual) and establishing relationships, which are often compromised among children exposed to institutional care. These skills are commonly referred to as “soft” or “interpersonal” skills and can be categorized into sub-skills such as social presentation, social scanning, and social flexibility (Taborsky & Oliveira, 2012). For socially vulnerable children, the development of adequate social skills is essential for creating positive social-emotional learning. Having good social skills is necessary for creating a positive environment in social situations and is crucial for emotional control, self-image, and positive thinking, which are important aspects of social-emotional learning (Bleidorn et al., 2016).

Children exposed to emotional and behavioral challenges, particularly those growing up in socially vulnerable environments such as child protection and welfare institutions, often struggle to master skills like empathy, social practice, and flexible interaction, highlighting the significance of social competence (Gresham et al., 2000). The concept of social competence is complex, making it challenging to establish a universally accepted definition for social competence, given its complexity and variability depending on the situation, especially when applied to a vulnerable population (Gundersen & Ziliak, 2014; Monnier, 2015). The lack of a universally recognized definition of social-emotional competencies complicates the task of successfully incorporating these skills into educational and welfare programs. Wu and Lingfei (2008) emphasize the teachable nature of social skills and their relevance in different situations, pinpointing factors such as social appearance, observation, and flexibility.

Emotional skills, also known as emotional competence, are a set of important abilities for mental well-being, such as self-awareness, empathy, and regulating emotions. Voronov and Weber (2016) highlight the significance of emotional competence in institutional settings, stressing the crucial role of proper emotional expressions for social integration in these environments. Research on improving emotional intelligence, like the work done by Wood and Neal (2007) and Hoemann et al. (2021), emphasizes the advantages of acknowledging and managing emotions. They propose that emotional proficiency helps in building resilience, personal development, and academic achievement. Portela-Pino et al. (2021) and Rupande (2015) emphasize the significance of emotional intelligence for students, promoting its contribution to overall growth. While different studies use the terms emotional competence and emotional intelligence interchangeably, the current study primarily used CASEL’s competency domain to maintain conceptual consistency.

The 2004 Punjab Destitute and Neglected Children Act requires *CP&WB* to provide services such as child welfare, foster care, and protective investigations to at-risk children. This action is in line with UNICEF’s child protection guidelines, tackling intricate problems like abuse and trafficking (Abbas, 2024; Jabeen, 2016).CP&WB Gujranwala, similar to other organizations, has an important impact on developing social and emotional skills in a structured, encouraging setting, especially for children from underprivileged backgrounds with minimal access to social and emotional development opportunities (Nayab et al., 2025).

Reich (2008) and Berrick et al. (2016) suggest incorporating social and emotional skills in child welfare decisions, promoting a strengths-focused method emphasizing resilience and personal strengths. The skills of self-awareness, social awareness, and responsible decision-making are essential for students to thrive and achieve success (Chernyshenko et al., 2018). Ross and Heath (2002) assert that school psychologists play a critical role in addressing the social and emotional needs of children facing behavioral challenges. Casey (2012) stresses the significance of teaching social skills, especially for students dealing with emotional difficulties, promoting a model that enhances students’ resilience and emotional intelligence.

It cannot be emphasized enough how crucial it is to nurture social and emotional skills in *CP&WB Gujranwala*, as they are vital for the overall development of children dealing with challenges. Multiple research studies support the beneficial effects of these abilities on both academic and life outcomes (Chernyshenko et al., 2018; Ross & Heath, 2002). Exploring these abilities in the framework of *CP&WB* provides an understanding of how institutional settings can support the development of overlooked children. Hence, a thorough examination of social and emotional abilities at *CP&WB Gujranwala* is crucial not only for comprehending the requirements of these children but also for improving support initiatives, rendering the research “An Analysis of Social and Emotional Skills of Students at Child Protection and Welfare Bureau Gujranwala” pertinent and timely.

These results highlight the need for continued study and aid in understanding the emotional and social growth of the neglected students inside the institution. The objectives of the study were as follows: (a) To analyze the levels of social and emotional skills among the students enrolled at *CP&WB Gujranwala*, (b) To examine the difference in social and emotional skills levels between students with shorter institutional stay (less than six months) and those with longer institutional stay (six months and more) at *CP&WB Gujranwala*, (c) To investigate the relationship between institutional stay duration and students’ social and emotional skills at *CP&WB Gujranwala.*

Objective (b) focuses on group-based differences, and objective (c) examines the continuous relationship between duration of stay and social-emotional skills development. Both objectives (b) and (c) address different analytical perspectives and provide complementary insights into the relationship between institutional stay duration and students’ social and emotional development.

### Research questions

- What is the level of social skills among students at *CP&WB Gujranwala?*
- What is the level of emotional skills among students at *CP&WB Gujranwala*?
- Are there any differences in social skills among students based on institutional stay at *CP&WB Gujranwala* (less than six months / six months or more)?
- Are there any differences in emotional skills among students based on institutional stay at *CP&WB Gujranwala* (less than six months /six months or more)?

On the basis of these questions, the researcher hypothesizes that there is a significant difference in social skills among students based on institutional stay at *CP&WB Gujranwala* (less than six months /six months or more). It is hypothesized that there is a significant difference in emotional skills among students based on institutional stay at *CP&WB Gujranwala* (less than six months /six months or more). It is also further hypothesized that there is a significant relationship between the duration of institutional stay (less than six months /six months or more) and students’ social and emotional skills at *CP&WB* in *Gujranwala*.

The researcher used non-directional hypotheses due to the absence of predetermined direction and the limited availability of evidence in the local context.

### Significance of the Study

This research holds considerable importance in advancing our comprehension of social and emotional competency levels among students within child welfare environments, specifically in relation to the *CP&WB Gujranwala*. It offers a distinctive perspective on how systematically organized institutional assistance exerts a favorable influence on the well-being of these at-risk children. Through the analysis of variables such as self-regulation, empathy, and social interaction across different durations of institutional stay, the investigation elucidates that prolonged residential periods are significantly associated with higher levels of skill acquisition, thereby emphasizing the critical nature of extended, supportive interventions for marginalized youth. This inquiry adds to the sparse body of literature concerning the institutional influence on the high level of social-emotional skills of youth in Pakistan, advocating for sustained and evidence-driven initiatives within child welfare systems aimed at nurturing these essential competencies in underserved populations.

## Methods

### Study type

The current study used a descriptive, sequential exploratory mixed-methods research design that consisted of two phases. In Phase I (qualitative), the researcher conducted semi-structured interviews with teachers. In Phase II (quantitative), the researcher collected data from students through a questionnaire. A mixed-method approach was considered appropriate for obtaining a comprehensive understanding of students’ social and emotional skills. This approach provides a brief and clear explanation of the importance of research design for triangulation in preserving the validity and reliability of the results (Aramo-Immonen, 2013; Oranga, 2025). The researcher utilized a cross-sectional comparative design for the quantitative part of the study. The qualitative findings of Phase I helped the researcher develop and adapt the quantitative instrument, ensuring contextual relevance and alignment with the study objectives.

The researcher used the CASEL framework as an organizing framework to categorize social and emotional skills (self-awareness, social awareness, self-management, relationship skills), to structure teachers’ interviews, and to interpret students’ questionnaire results, rather than as a program evaluation tool.

## Study sample

The study was conducted with a universal sample including the entire population from the *CP&WB* in *Gujranwala*, involving all 105 students enrolled in the institution and seven teachers who work closely with them. The researcher adopted this approach to ensure comprehensive data collection and to eliminate sampling error. Universal sampling provides a more reliable and accurate reflection of the institutional context than a subset sample could offer and reflects the total ecological reality of the research site (Besekar et al., 2024; Antoniou et al., 2024).

In the qualitative Phase (I), the researcher included all seven teachers who were directly in contact with students daily for at least six months because of their experience with students. In the qualitative phase, the researcher focused on getting a deep understanding rather than general results. The teachers provided experiential knowledge for understanding students, and their insights guided the researcher in adapting the questionnaire. Each teacher was responsible for about 15 students, and this student-to-teacher ratio is normal for the institutional structure of CP&WB. Although the teacher sample (*n* = 7) is smaller than the student sample (*n* = 105), this sample size reflects the logic of the sequential exploratory design. The qualitative phase prioritizes depth and meaning-making, while the quantitative phase ensures generalizability. Such imbalances are consistent with mixed method strands, where complementarity of phases is prioritized over numerical symmetry (Creswell & Plano Clark, 2023; Schoonenboom & Johnson, 2017). Furthermore, the limited availability of teachers compared to students is a common practical consideration in educational research (Hanafi, 2021; Koc & Celik, 2015), making the chosen ratio both realistic and methodologically sound.

This comprehensive sampling strategy aimed to capture a full spectrum of social and emotional skill development among students across different ages, classes, and lengths of stay. The age of students ranged from five to sixteen years, representing a wide developmental spectrum. Differences in social and emotional skills across age groups may exist. In this study, the researcher’s aim was not to compare the social and emotional skills of the students across different age groups, and age-wise comparisons were not conducted due to the limited subgroup sizes. The researcher acknowledges it as a limitation of the study.

Teachers provided contextual insights into students’ progress, while the entire student body’s responses enabled statistically meaningful analysis of social and emotional competencies within the *CP&WB Gujranwala* environment. For the quantitative part, the researcher categorized students into two groups (less than six months and six months or more) based on their duration of institutional stay to examine differences in social and emotional skills. Insights from Phase I teachers’ data led the researcher to categorize students into two groups. Teachers reported noticeable changes in students’ behaviors after about six months of stay in the institution. This confirms the six-month period as a critical adjustment phase. This informed the grouping of students for comparison in the quantitative Phase II.

In previous studies, it was also observed that within a single academic semester of about six months, students’ behavior shifts noticeably; teachers are reliable primary observers and capable of observing these changes within short-term instructional windows (Antoniou et al., 2024; Wigelsworth et al., 2022). Unlike formal school settings, the *CP&WB Gujranwala* is an institution where vulnerable children live and learn together. Social and emotional skills are not taught as a separate subject. Instead, students learn these skills through daily life activities. They learn during morning assembly, group study, classroom practices, and playtime. They learn problem-solving, sharing, empathy, self-awareness, teamwork during supervised play. Teachers also model good behavior.

## Instruments and measures

This study was conducted in two phases. In Phase I, the researcher formulated a face-to-face semi-structured interview protocol, based on existing literature, including Naz et al. (2022), and Ruslin et al. (2022). The researcher gave the teachers an orientation before the interviews. The purpose of this orientation was to familiarize them with the terminology related to social and emotional skills (relationship-building, self-awareness, emotional regulation, etc.). The researcher did not administer any competency test before the interview, and acknowledged this omission as a limitation of the study. Teachers were chosen as respondents because they provide critical insights into students’ social and emotional skill development. Face-to-face semi-structured interviews were deemed the most suitable. Semi-structured interviews provide a means for researchers and interviewees to collaboratively elucidate adequacy and receptiveness (Irvine et al., 2013). The purpose of the interview protocol was to explore the factors influencing changes in students’ social and emotional skills and to ensure the accuracy of the data collected. The protocol consisted of questions related to students’ social skills, emotional skills, institutional effect, and changes in students’ behaviour with the passage of time in institutional stay. Open-ended questions were addressed in interviews. Sample questions included: “Would you explain the social behaviours you observed in children at CP&WB Gujranwala?” “What challenges do children face when making friends or sharing with others?” “Would you explain the emotional behaviours you observed in children?” “In your experience, “have you noticed any change in children’s behaviour over time? If yes, what kind of changes did you observe?” Additional questions explored students’ emotional behaviours, adjustment patterns, and changes associated with institutional stay duration. Informed consent was obtained from all the teachers prior to data collection. The researcher conducted all interviews in person, ensuring that all respondents were pre-informed about the recording of the interviews. Each interview lasted approximately 30 minutes.

Recording interviews is considered an acceptable method of obtaining interview data more efficiently. Handwritten notes taken during an interview can be relatively inaccurate, causing the researcher to overlook several important points. By recording the interview, the interviewer can concentrate on the verbal cues and the conversation content, producing an accurate transcript (Jamshed, 2014).

In Phase II, the researcher adapted the questionnaire to employ it as a tool for data collection due to its cost-effectiveness, provision of anonymity, time-saving attributes, and recognition as an optimal method for mitigating interviewer bias (Gjersing et al., 2010; Sousa et al., 2017; Walliman, 2021). The researcher adapted the questionnaire on the basis of themes identified in Phase I. Teachers frequently mentioned some behaviours such as sharing, anger control, and seeking help. These themes mapped onto CASEL’s four competency domains: self-management, self-awareness, social awareness, and relationship skills. Although the original instrument was not specifically developed according to CASEL’s framework, the adapted items demonstrated conceptual alignment with CASEL’s competency domain and were therefore used to organize the constructs analysed in the current study.

The questionnaire comprised two main sections: an introductory cover letter and demographic characteristics (class, age, and stay duration). Section two consisted of items related to social and emotional skills adapted from previously validated instruments, the “Social Emotional Development” scale developed by Brenchley (2017). The original instrument was publicly and freely available as an open-access dissertation. Minor modifications were made to the wording of items to ensure cultural relevance and clarity for the research population. The original scale consisted of 36 items. The researcher selected 28 items that were most appropriate and relevant. The selected items were categorized into social and emotional skill domains based on their conceptual alignment with CASEL’s competency domains. The researcher translated all the items into Urdu for cultural appropriateness, using backward and forward translation methods with the help of bilingual experts, followed by the established guidelines of Cruchinho, López-Franco, Capelas, Almeida, Bennett, Miranda da Silva, and Gaspar (2024). This process enhances validity, minimizes semantic bias, and preserves the intended meaning of the constructs (Adeel, Imran, & Syeda, 2025; Jabeen & Maqsood, 2023). The original instrument utilized a visual thumb clip art scale with three thumb signs (thumbs up, thumbs down, and thumbs sideways). The researcher adapted the scale into a three-point frequency scale to ensure comprehension among young participants in this context of study. Studies suggest that such scales are commonly used in educational and psychological research to reduce the cognitive load in children for accurate response (Krosnick, 2018; Likert, 1974; Revilla et al., 2014). The adapted questionnaire was reviewed by experts in education and psychology, who evaluated the relevance, clarity, and appropriateness of the items to ensure that the items were appropriate for the context of the CP&WB institution in Gujranwala. The reliability of the tool was assessed by using Cronbach’s alpha. Cronbach’s alpha results (α = 0.976) indicated excellent internal consistency.

The researcher used a three-point frequency scale to determine the level of students’ social and emotional skill levels. The researcher physically administered the questionnaire among students and read it aloud so that students with weak reading skills could understand and respond correctly to the items. Mean scores were categorized into low, moderate, and high levels using the range method. A three-point Likert scale was employed (1 = Never, 2 = Sometimes, and 3 = Always). Such simplified response scales are considered appropriate for younger population in educational and psychological research **(**Chakrabartty, 2023; Krosnick et al., 2015; Revilla et al., 2014). The cut-off interval was calculated by dividing the score range by the number of levels (3 − 1). Accordingly, mean scores of 1.00-1.66 were considered low, 1.67–2.33 moderate, and 2.33-3.00 high. According to Alem (2020), Marshall and Jonker (2010), and Alkharusi (2022), equal-interval ranges for descriptive interpretation are the most fundamental and frequently used psychometric tools in educational and social sciences research. Informed consent was sought from the District Supervisory officer in charge of the Child Protection and Welfare Bureau institution in Gujranwala before the data collection. Data were collected at a single point in time from students.

### Data analysis

Data analysis was conducted in two phases, integrating both quantitative and qualitative methods for a robust understanding of findings. Qualitative Analysis (Phase I): Qualitative data was analysed using thematic analysis following Braun and Clarke’s six-step approach (Braun & Clarke, 2006; Cernasev & Axon, 2023; Kiger & Varpio, 2020; Naeem et al., 2023; Sandhiya & Bhuvaneswari, 2025). Thematic analysis was selected because it allows for an in-depth understanding of teachers’ perceptions regarding students’ social-emotional skill development. The researcher analysed the data using inductive thematic analysis following the Braun and Clarke (2006) six-step framework. The researcher identified the themes at the semantic level, focusing on what participants explicitly said rather than interpreting hidden meanings (DeJonckheere et al., 2024). The researcher transcribed the interviews at the first stage. Then the researcher read the transcripts repeatedly to achieve familiarity with the data at the second stage. At the third stage, the researcher generated the initial codes, and at the fourth stage, the researcher grouped all the similar codes to form the themes. At the fifth stage, the researcher reviewed the themes multiple times to ensure consistency and credibility and refined all the themes. At the sixth stage, the researcher named all the themes and interpreted them.

To enhance the trustworthiness and credibility of the qualitative findings, the identified codes, categories, and themes were reviewed and discussed with the research supervisor until consensus was achieved. Any disagreements or ambiguities in coding and theme interpretation were resolved through discussion and refinement. These supervisory discussions served as a verification strategy to strengthen the rigor and validity of the qualitative analysis.

A thematic analysis of interview data provided depth to the quantitative findings, uncovering themes such as social stability, emotional transformation, and adaptation to structured environments. Teacher interviews highlighted variations in students’ adaptive behaviors, emphasizing how individual backgrounds and past experiences shape students’ social and emotional development. These qualitative insights allowed for a layered understanding of the unique factors influencing each student’s growth. This approach provides a holistic understanding of students’ social and emotional skills at *CP&WB Gujranwala*, enabling triangulation and validation of findings (Jones et al., 2023). It also offers a subtle analysis, revealing factors influencing their skills and potential intervention strategies (Evans et al., 2017).

Quantitative Analysis (Phase II): The amount of information obtained through the questionnaire was investigated utilizing SPSS version 23 software. Descriptive and inferential statistics were applied to the survey data to examine the level of social and emotional skills and their relationships with the duration (less than six months / six months or more) of stay at *CP&WB Gujranwala*.

Furthermore, research indicates that the six-month stay duration in an institution is a critical adjustment period for students’ social and emotional skills (Barnhart, 2021; Durlak et al., 2011). Descriptive statistics outlined the prevalence and level of students’ social and emotional skills, while independent sample *t*-tests and Pearson correlation were used to determine the group differences and the association between stay duration at the institution and students’ social and emotional skills. The researcher performed Levene’s test for equality of variance before the independent sample *t*-tests to assess the assumption of homogeneity of variance and reported the appropriate results accordingly. Table 1 presents the themes and subthemes derived from the interview data collected from teachers.

**Table 1:** Themes and sub themes of interview data collected from teachers

Main Themes	Sub Themes
1) Social Stability and Interaction	Varied Difficulty in Social Stability - Differences in Adaptation Time - Initial Reluctance to Share - Challenges in Social Interaction - Differences in Playing and Making Friends
2) Emotional Attitudes and Transformation	Diverse Attitudes Based on Friends and Parents - Distinct Empathy and Sensitivity Levels - Training and Class Attendance Leading to Behavioral Changes - Formation of Friendships and Sharing - Positive Emotional Development
3)Adaptation and Change	Individual Differences in Adaptation - Improvement Over Time - Gradual Transformation Through Living in a Structured Environment

## Results

### Qualitative thematic analyses

#### Phase I

##### Social skills of students

Based on the insights provided by the participants, children face varying levels of social stability, which reflect differences in their ability to interact, cooperate, and adjust socially. Some are rescued from diverse backgrounds, while others excel in playing and making friends, and others struggle with sharing. Participants described inconsistencies in social and emotional skills development among students. Teachers noted that these differences among students’ social and emotional skills were influenced by their past experiences and limited opportunities for social learning. As per participants’ views:

*Every child has a varied level of difficulty when it comes to social stability; some youngsters get confused easily*,* while others take longer (Participant 1).*

*These kids have no desire to be around other kids; they are rescues from various backgrounds. They don’t even share their belongings. They first quarrel with one another (Participant 2).*

*Some kids are so adept that they play with their pals and make new friends. Some kids find it awkward to share these kinds of things (Participant 3).*

*These kids have different skills and are far smarter than our kids. They simply aren’t given the opportunity to reach their full potential (Participant 4).*

##### Emotional skills of students

According to the insights provided by the participants, children who had diverse kinds of friends and a parental background displayed distinct attitudes. Many children showed oversensitivity, difficulty with emotional regulation, and strong reactions to loss or separation. The process by which children show compassion differed based on the circumstances and their prior experiences. Teachers noted that some students displayed empathy. Some students were emotionally distressed and hyperactive when they first entered the institution. According to the participants’ views, students’ emotional responses were linked to their past trauma, unstable family environments, and changes in caregiving relationships. As per participants’ views:

*They appear to be quite sensitive. Then*,* a month later*,* it becomes evident that they have changed and no longer understand? They differ greatly in personality (Participant 1).*

*When these kids come to the institution*,* they are very hyper. Diverse kinds of kids are in diverse circumstances; some kids have issues at home. Some people struggle with parenting. Someone has financial issues. They are dealing with a wide range of issues. They want to hurt themselves at first (Participant 2).*

*Before coming here*,* these youngsters had a fantastic time and experienced their parents’ affection. However*,* misfortune befalls them*,* and their parents part ways or pass away*,* leaving these children living here and receiving care. Those who live with friends behave slightly differently from employees and teachers (Participant 5).*

##### Social and emotional skills improved in the institute

Participants observed that students’ social and emotional skills improved over time due to structured activities, training, and interactions in the institution. Teachers noted significant changes in their behavior; they formed friendships, shared belongings, and developed affection for each other. Each child adapted differently; most showed improvement in behavior, cooperation, and emotional regulation. The speed of these modifications is influenced by former backgrounds and personality attributes. Teachers noted that residing in a structured, supportive institutional environment fostered social integration and emotional well-being in students. As per participants’ views:

*Every child is different; they all perceive and react to the world in various ways. 45% of the time*,* these behaviors improve over time as they adjust to their surroundings (Participant 1).*

*The kids approach their teacher. They attend class and give it a go. They are trained. They start playing together*,* and these kids develop close bonds. They undergo a lot of transformation. They begin to share things and develop positive feelings for one another (Participant 6).*

*After living in this setup*,* kids progressively transform and begin interacting with others by playing*,* mingling*,* sharing*,* and coexisting. These kids will undergo adjustments if they reside here. It causes their behavior to improve from what it was before (Participant 7).*

### Quantitative data

#### Phase II

Table 2 shows the demographic variables of students. 36.4% participants’ ages were between 8 and 10 year, which was the highest number (39), while 9.3% participants’ ages were between 14 and 16 years, which was the lowest (10) 0.72% students belonged to Prep-One class, which was the highest number of students (77), while 6.5% of students belonged to Four-Five class, which was the smallest group (7). 40.2% participants’ stay duration was less than six months in the institution, while 57.9% of participants’ stay duration was six months or more.

**Table 2:** Demographic analyses of students

Variables	Frequency	Percent
*1*		
5year-7year	34	31.8%
8year-10year	39	36.4%
11year-13year	22	20.6%
14year-16year	10	9.3%
*Students’ Class*		
Prep-One	77	72%
Two-Three	15	14%
Four-Five	7	6.5%
*Students’ Stay Duration*		
Less than six months	43	40.2%
Six months & more than six months	62	57.9%

Overall findings revealed that a majority of the students were between the ages of 8 and 10 years. A large number of students were enrolled in Prep-One grade, which suggests that many students were at an early stage of formal schooling. This is relevant for understanding the development of basic social and emotional skills. The majority of students’ institutional stay duration was six months or more. This is important because the length of students’ institutional stay may influence their social and emotional skills.

Table 3 descriptive statistics results showed the level of social skills among students at CP&WB Gujranwala. Findings revealed that across all the variables of social skills, students demonstrated a moderate to high level of social skills. 65*%* (*M* = 2.6, *S.D* = 0.467) of students responded “Always,” which consistently demonstrated a high level of social skills; 35% of students responded “sometimes,” and approximately 0.5% students chose the response “Never.”

**Table 3:** level of Social Skills among Students at CP&WB gujranwala

Statements	Never (%)	Sometimes (%)	Always (%)	M	S. D
I don’t hurt other kids.	7.5%	44.9%	45.8%	2.39	0.627
I raise my hand when I have a question.	00	45.8%	52.3%	2.53	0.501
If an activity gets cancelled I don’t complain.	00	43.0%	55.1%	2.56	0.498
My work is not messy.	00	36.4%	61.7%	2.62	0.485
I like to share my toys.	00	34.4%	63.6%	2.64	0.480
I can join in games other kids play.	00	32.7%	65.4%	2.66	0.473
I invite kids to play with me.	00	32.7%	65.4%	2.66	0.473
My teachers like me.	00	12.1%	86.0%	2.87	0.330
People at school care about me.	00	33.6%	64.5%	2.65	0.476
There are many people I can talk to if I have a problem.	00	37.4%	60.7%	2.61	0.487
School is wonderful place.	00	34.6%	63.6%	2.64	0.480
People are happy at school.	00	15.9%	82.2%	2.83	0.370
I like to help my teacher.	00	15.9%	82.2%	2.83	0.370
I am a good listener to other kids.	00	56.1%	42.1%	2.42	0.497
*Social Skills*	0.5%	34.5%	65.0%	2.6	0.467

Level of Social Skills among Students at CP&WB Gujranwala

*M* Mean, *S.D *Standard Deviation

Item-level results indicated strong school connectedness and peer interaction, with high mean scores for items such as “teachers like me” while the mean score was 2.87( *SD* = 0.33), “people are happy at school” (*M* = 2.83, *SD* = 0.37), and “people at school care about me” while the mean score was 2.65 (*SD* = 0.48). Similarly, social participation behaviors, such as joining games, the mean score was 2.66 (*SD* = 0.47), and inviting peers to play, the mean score was 2.66 (*SD* = 0.47), showed high scores. In contrast, comparatively lower mean scores were observed for “not hurting other kids” (*M* = 2.39, *SD* = 0.63) and “being a good listener” (*M* = 2.42, *SD* = 0.50), suggesting variability across social skill domains.

Overall findings revealed that across all variables of social skills, the majority of students reported positive behaviors and attitudes. 65*%* (*M* = 2.6, *SD* = 0.467) of students consistently exhibited positive social skills such as engaging in peer interactions, playing cooperatively, and helping others. Notably, 35*%* of students demonstrated moderate interpersonal abilities. However, approximately 0.5*%* of students struggled with certain aspects of social skills.

Table 4 descriptive statistics results showed the level of emotional skills among students at CP&WB Gujranwala. Findings revealed that across all the variables of emotional skills, students demonstrated a moderate to high level of emotional skills. 65*%* (*M* = 2.6, *SD* = 0.477) of students responded “Always,” which consistently demonstrated a high level of emotional skills; 35% students responded “sometimes,” and approximately 0.5% students chose the response “Never.” Higher mean scores were observed for items reflecting positive self-concept and supportive relationships, including “teachers are helpful” while the mean score was 2.86(*SD* = 0.34) and “teachers notice when I do my best work” while the mean score was 2.80 (*SD* = 0.39). Similarly, students reported favorable self-perceptions, such as “liking how they look” while the mean score was 2.64 (*SD* = 0.48) and “being a good kid” while the mean score was 2.61(*SD* = 0.49).

**Table 4:** Level of emotional skills among students at CP&WB gujranwala

Statements	Never (%)	Sometimes (%)	Always (%)	M	S. D
I use my words to tell someone if I’m angry.	00	44.0*%*	53.0*%*	2.54	0.500
I tell people that I’m happy.	00	43.0*%*	55.1*%*	2.56	0.498
I smile a lot.	00	43.0*%*	55.1*%*	2.56	0.498
I can tell someone I’m upset without yelling.	00	44.9*%*	53.3*%*	2.54	0.500
My family cares about me.	00	44.9*%*	53.3*%*	2.54	0.500
People like me even when I’m having a bad day.	00	43.9*%*	54.2*%*	2.55	0.499
I like to learn new games even if they seem hard at first.	00	47.7*%*	50.5*%*	2.51	0.502
I am not great at every game but I try.	00	44.9*%*	54.2*%*	2.55	0.499
I like how I look.	00	34.6*%*	63.6*%*	2.64	0.480
I am a good kid.	00	37.4*%*	60.7*%*	2.61	0.487
Good things happen to me.	00	38.3*%*	59.8*%*	2.60	0.490
Teachers are helpful.	00	13.1*%*	85*%*	2.86	0.341
My teachers notice when I do my best work.	00	18.7*%*	79.4*%*	2.80	0.394
I like to help kids when they are sad.	00	45.8*%*	52.3*%*	2.53	0.501
*Emotional Skills*	0.5	*34.5%*	65.0%	2.6	0.477

*M *Mean, *S.D *Standard Deviation

Emotional expression and regulation skills, including “expressing anger verbally to tell someone if I’m angry”, the mean score was 2.54(*SD*=0.5), and communicating upset feelings calmly, “telling someone I’m upset without yelling”, while the mean score was 2.54(*SD*=0.5), showed relatively consistent but slightly lower mean scores, indicating some variability across emotional skill domains.

Overall findings of the table revealed that across all variables, 65*%* (*M* = 2.6, *SD* = 0.477) of students consistently demonstrated emotional skills. Notably, 34.5*%* of students demonstrated emotional awareness, regulation, and resilience. However, approximately 0.5*%* faced challenges in managing emotions, with difficulties in areas such as anger management, prolonged sadness, and consistent kindness. The data reveal that students at CP&WB Gujranwala demonstrate varying levels of emotional skills.

Table 5 shows that the results of the independent samples *t*-test revealed a significant difference in social skill means between the two groups, *t*(103) = -13.18, *p* < .001. This result indicates that students’ social skill levels differed significantly based on their institutional stay.

**Table 5:** Significant differences in students’ social skills based on institutional stay duration

		F	Sig.	t	df	Sig. (2-tailed)
Social Skills	Equal variances assumed	3.401	0.068	-13.183	103	0.001

Significant

Table 6 shows that the results of the independent samples t-test revealed a significant difference in emotional skill means between the two groups, *t* (103) = -15.19, *p* < .001. This result indicates that students’ emotional skill levels differed significantly based on their institutional stay.

**Table 6:** Significant differences in students’ emotional skills based on institutional stay duration

		F	Sig.	t	df	Sig. (2-tailed)
Emotional Skills	Equal variances assumed	2.492	0.118	-15.190	103	0.001

Significant

Table 7 shows the results of Pearson correlation analyses examining the relationship between institutional stay duration and students’ social and emotional skills. The correlation value between students’ institutional stay duration and social skills is 0.772, which indicates a strong positive correlation. The *p*-value is < 0.01, which means the relationship is statistically significant. The correlation value between students’ institutional stay and emotional skills is 0.781, which indicates a strong positive correlation. The *p*-value is < 0.01, which means the relationship is statistically significant. These findings indicated that there is a significant association between institutional stay duration and students’ social and emotional skill levels.

**Table 7:** Correlations between the institutional stay duration and students’ social and emotional skills

Students’ institutional stay duration	Students’ institutional stay duration	Social Skills	Emotional Skills
	1		
Social Skills	0.772^**^	1	
Emotional Skills	0.781^**^	*0.910* ^**^	*1*

**. Correlation is significant at the 0.01 level (2-tailed)

## Discussion

Child Protection and Welfare Bureau (CP&WB) Gujranwala is a significant institution concerned with protecting children’s rights and ensuring their well-being. To enhance the efficacy of child welfare protection initiatives, *CP&WB Gujranwala* engages in partnerships with multiple organizations and interested parties (Apeli & Kisimbii, 2020; Nayab et al., 2023). The social and emotional growth of students is vitally important since it has a direct impact on their overall development, well-being and ability to adapt in socially vulnerable contexts (Nayab & Sarwar, 2025). This is particularly important because children’s learning and performance are greatly impacted by their social and emotional abilities (Chernyshenko et al., 2018; Ross & Heath, 2002). The results of the present study contribute to the growing body of literature that highlights the importance of structured institutional support for promoting social and emotional skill development among vulnerable students.

Teachers highlighted the variability in children’s social skills, indicating diverse adaptation levels. Additionally, emotional skills, like empathy, were observed to differ based on the diversity of parents and peers. Research has shown that structured social skills training, including instructions, practice, and review, has been found to considerably increase social skills and friendship-making ability (Lecroy & Beker, 2014). Furthermore, teachers’ insights emphasize the importance of a structured and safe environment for students’ social and emotional skills. This is consistent with the previous research reporting that children recognize and express their feelings appropriately in emotionally safe and structured environments (Harjunmaa et al., 2023; Shean & Mander, 2020; Veale et al., 2023). This could be related to research that reported that social and emotional skills levels among institutionalized children with unique challenges considerably increase within a structured environment (De Mooij et al., 2020; Moretti et al., 2024). These findings align with CASEL’s competencies of self-management, relationship skills, and social awareness, and suggest that structured institutional routines and repeated social interactions may help students to gradually develop social and emotional skills.

The demographic profile of students showed a variety of age groups, classes, and their institutional stay durations. Notably, over half of the students’ institutional stay duration was six months or more. Descriptive statistics indicated generally positive social and emotional skill levels. The students reported positive behavioral tendencies, indicating a nurturing environment. The significant positive responses in various scenarios, like sharing toys and participating in activities, suggested students’ high levels of social and emotional skills. These findings align with the studies reporting that institutional environments influence students’ social and emotional skill levels (Bjugstad et al., 2025; Chan & Lam, 2023; Fang et al., 2025). These behavioral tendencies may be influenced by consistent supervision, peer involvement, and planned daily activities within the institutional environment, all of which can help students practice social interaction and emotional adjustment skills.

The statistical analysis further confirmed a significant association between institutional stay duration and students’ social and emotional skills levels. Students with longer institutional stays tend to exhibit higher levels of these skills. These findings are consistent with studies reporting that supportive environments are associated with enhanced social and emotional skills in students, particularly in the context of vulnerability (Carriedo et al., 2024; Chowa et al., 2023; Hébert, 2022). The results indicate that the *CP&WB* plays a crucial role in students’ well-being. The positive changes observed in students’ behaviors and attitudes with longer institutional stay duration underscore the effectiveness of the institute’s structured environment. These findings suggest that longer exposure to organized institutional routines and emotionally supportive relationships helps students adjust socially and emotionally over time. The correlation analysis establishes a strong positive relationship between the longer institutional stay duration and both social and emotional skills. This emphasizes the importance of a longer stay for maximizing students’ well-being. The findings suggest that prolonged stays in supportive environments positively influence students’ social and emotional skills levels. From the perspective of CASEL’s competency domains, prolonged institutional exposure may provide opportunities for practicing social and emotional skills. Furthermore, these findings can be interpreted through Bronfenbrenner’s Ecological Systems Theory, according to which a long stay in a structured environment influences students’ social and emotional skills (Yang & Oh, 2024).

However, findings of the current study appear to contradict other research. Neglected and destitute children who grow up under institutional care often face many challenges and find it difficult to understand and control their emotions (Ismayilova et al. 2023a, b; Nduku et al. 2022; Yin 2024). Many international studies, such as Moretti et al. (2024); Rohanachandra et al. (2022); Ismayilova et al. 2023a, b), find that children who grow up under institutional care struggle with behavior problems, peer pressures, and social and emotional difficulties. These students find it difficult to communicate with others, show empathy, share, and cooperate (Lemos et al., 2021; Simão et al., 2025). Research found that these students may feel lonely, sad, and anxious (Xiong et al., 2023). The variation between the findings of the present research and those of previous studies could be attributed to contextual differences such as institutional structure, caregiving quality, duration of institutional stay, cultural environment, and availability of social and emotional support systems. These differences between studies on students social and emotional skills may be due to different variables such as how long they stay in institutional care, their background, how many times they changed the institution, and the country where the institution is located (Almas et al., 2020; Debnath et al., 2023; Kaur et al., 2018; Lamm et al., 2018; Li et al., 2019). Overall, these studies showed that neglected and destitute children living under institutional care need a structured, safe environment and consistent social and emotional support.

## Conclusion

The current study examined the social and emotional skills of students enrolled at the CP&WB Gujranwala. The researcher used a sequential exploratory mixed-methods research design. Teachers’ insights were integrated with students’ self-reported responses. The findings suggested that a considerable portion of students at the Child Protection and Welfare Bureau reported moderate to high levels of social and emotional skills, including coordination, emotional expression, empathy, and social interaction.

Students with longer institutional stay durations reported higher levels of social and emotional skills in comparison with students with shorter institutional stay durations. Teachers’ observations further support the notion that a structured institutional environment is associated with students’ social and emotional learning. The diversity in students’ social and emotional skills learning highlights the role of structured routines, guidance, and emotional support within the institutional environment.

Findings of the current study reported that CP&WB Gujranwala provides a supportive and structured environment for children. On the contrary, findings of some existing research documented that difficulties with emotional control, lack of empathy, cooperation, sharing, communication, sadness, and loneliness are associated with students who are under institutional care. The findings of the current study do not negate these risks but rather indicate that structured institutional practices may predominantly buffer against vulnerability among children. The current research was cross-sectional, the data were self-reported, and it was conducted within a single institution, which limits generalizability.

### Limitation

There are some limitations in the current research that must be acknowledged. The current research was conducted with teachers using a qualitative data collection tool and with students using a quantitative data collection tool. Students were not interviewed in the current research, which can further restrict an in-depth understanding of their behaviors. A cross-sectional design was employed, which further prevents conclusions about students’ skill levels. In this study, students were from wide developmental age groups (5–16 years) with a small sample size in age subgroups. Social and emotional skills develop differently across childhood to adolescence. In the current study, the researcher did not compare students’ skills within different age groups due to the small sample size of students’ age subgroups. A comparison could be performed in future research to analyze the difference in social and emotional skill development among students using a large and age-stratified sample. Students institutional stay duration and its association with students social and emotional skills levels were examined as a categorical grouping variable. It could be examined through a longitudinal study. Moreover, students were not given any treatment; an experimental and observational study design can provide more in-depth detail about the variability among students’ social and emotional skill levels. The qualitative data were based on self-reported responses, which can become biased and may be influenced by students’ social conformity. The current study was conducted in a single institute of CP&WB, which can further limit the generalizability of findings. Teachers were not tested on their understanding of social and emotional learning terminology, which is acknowledged as a limitation of the study. Despite all these limitations, the current research contributes to the limited empirical literature available on destitute and neglected children’s social and emotional skills under institutional care in the context of Pakistan. Teachers’ perspectives provides the foundation to understand the students’ social and emotional challenges within the CP&WB institutional care. The integration of both qualitative and quantitative data sets, teachers’ perspectives, and students’ self-reported data provides a holistic view for understanding of social and emotional skills and their association within the institutional settings.

### Recommendations

Several recommendations are proposed for future researchers based on the findings and limitations of the study.

Future research may conduct longitudinal studies monitoring the same students over time at the *CP&WB Gujranwala* to assess the increase in social and emotional skill level associated with long institutional stay duration. Additionally, future research should examine the manner in which the socio-emotional learning of students is influenced by external variables such as family participation, community involvement, and societal perspectives. Cross-cultural studies are also recommended to enhance generalizability and to gain insights into how cultural factors may influence the effectiveness of interventions and the learning of social and emotional skills. Lastly, further research may investigate the role of teachers’ training and structured social and emotional learning interventions in promoting the well-being of students within the *CP&WB* institution in *Gujranwala.*

## Data Availability

No datasets were generated or analysed during the current study.

## Abbreviation

CP&WB: Child Protection and Welfare Bureau
